# Community Strengthening in Urban Neighborhoods: Psychosocial Dynamics in Vulnerable Contexts

**DOI:** 10.1002/jcop.70058

**Published:** 2025-12-09

**Authors:** Alba Zambrano Constanzo, Vaite Trujillo, Mauricio García Ojeda, Francisca Román Mella

**Affiliations:** ^1^ Department of Psychology Universidad de La Frontera Temuco Araucanía Region Chile; ^2^ Department of Social Sciences Universidad de La Frontera Temuco Araucanía Region Chile

**Keywords:** community psychology, community psychosocial dynamics, community strengthening, participatory evaluation, dinámica psicosocial comunitaria, fortalecimiento comunitario, evaluación participativa, psicología comunitaria

## Abstract

This study aimed to understand the local particularities and common aspects of community psychosocial dynamics in different neighborhoods of the city of Temuco. These neighborhoods share conditions of social vulnerability and established a collaborative agreement with the research team. A participatory evaluation was conducted using mixed methods and participatory data production methodologies. The study involved key informants, members of organizations, and residents from each neighborhood. Both an ongoing analysis of each neighborhood and an integrated analysis were performed to address the study's objectives. In all three neighborhoods, a strong link was identified among their psychosocial dynamics, historical origins, and organizational trajectories associated with ensuring subsistence and urban development. Currently, there is evidence of organizational capacity, a sense of community, and social capital, particularly among older residents, who play more active roles in local organizations and demonstrate greater involvement in community life. However, empowerment is concentrated in individuals holding leadership roles and remains limited within organizations. Although a sense of community prevails, there are no overarching objectives that unite residents in shared goals for the common good. The level of political engagement in organizational life and interactions with public policy is low. In one of the neighborhoods, some female leaders exhibit a broader sociopolitical understanding, fostering a critical stance toward clientelistic relationships with political actors. The participatory evaluation process has shown positive effects on the neighborhoods’ participatory dynamics, alongside other advancements in community strengthening.

## Resumen

El presente estudio se propuso comprender las particularidades locales y aspectos comunes de las dinámicas psicosociales comunitarias de distintos barrios de la ciudad de Temuco. Estos barrios comparten condiciones de vulnerabilidad social y establecieron un acuerdo de trabajo conjunto con el equipo investigador. Se desarrolló una evaluación participativa, empleando métodos mixtos y metodologías participativas de producción de datos. Incluyó la participación de informantes claves, integrantes de organizaciones, y miembros de cada barrio. Se efectuó un análisis en progreso de cada barrio, así como un análisis integrado para dar respuesta a los objetivos del estudio. En los tres barrios aparece un estrecho vínculo entre sus dinámicas psicosociales con los orígenes de cada barrio y con trayectorias organizacionales asociadas a garantizar la subsistencia y al mejoramiento urbanístico. En la actualidad, se aprecia capacidad de organización, sentido de comunidad y capital social, especialmente entre las y los integrantes de mayor edad, quienes concentran roles más activos en las organizaciones, así como mayor involucramiento en la vida comunitaria. El empoderamiento se concentra en personas que ejercen roles de liderazgo y escasamente en las organizaciones. Si bien prevalece un sentido de comunidad, no hay objetivos transversales que integren a las y los vecinos en propósitos de alcance compartido asociados al bien común. Se aprecia escasa politización en la vida organizacional y en la relación con la política pública. En uno de los barrios, algunas lideresas tienen una comprensión sociopolítica más amplia, lo que favorece una actitud crítica ante las relaciones clientelares que tienden a prevalecer con actores políticos. El proceso de evaluación participativa muestra efectos positivos en la dinámica participativa de los barrios, así como otros avances en la línea del fortalecimiento comunitario.

## 1 The Challenges of Community Strengthening in Neoliberal Contexts

In Chile and Latin America, neoliberalism has been established as a societal model since the past century, transforming not only the economic system but also relationships, subjectivity, consumption and accumulation patterns, as well as the regulation of social life (Undurraga 2015). This model governs individual and collective subjectivity, establishing a regime that includes, among other elements, competition and distrust in the capacity for collective action to improve living conditions (Monbiot 2016). An exacerbated individualism negatively impacts social cohesion processes (Araujo and Martuccelli 2020) and hinders the construction of solidarity bonds, while also generating a “subjective emptying of the capacities to causally explain the problems of communal life“ (Báez 2020, p. 21). This depoliticizes a significant part of the problems and conditions that limit the development of individuals and communities.

This scenario challenges the positioning of community psychology, which views communities as active agents and producers of knowledge (Kagan et al. 2022). Therefore, in intentional processes of community mobilization, it is crucial to employ methodologies and strategies aimed at fostering reflection on the underlying logics of community problems while enabling co‐construction processes (Vázquez‐Rivera et al. 2021). This approach can contribute to strengthening the community's capacities for reflection and transformative action, promoting social cohesion and organization (Mesa and Restrepo 2020).

In the field of community psychosocial intervention, neoliberalism has fostered the implementation of social policies that do little to strengthen community capacities and instead promote targeting, the transfer of material resources, and assistance within a paternalistic or clientelist framework (Astete Cereceda and Vaccari Jiménez 2017). In most of these policies, the goal is not to transform the living conditions of communities but rather to generate adjustments that allow marginalized sectors to adapt to dominant dynamics. While these policies may contain, at a declarative level, elements of a community approach, in practice, they instrumentalize communities (Berroeta et al. 2019).

In Chile, neighborhoods with high levels of vulnerability have been the preferred site for public intervention through neoliberal social policies in areas such as security and poverty. According to Letelier et al. (2019), neoliberalism has positioned the neighborhood as an ideological device that has produced a closed conception of neighborhood life, characterized by elements such as restricted proximity relations, encapsulation and hyper‐localism, homogenizing ties, highly institutionalized and controlled organizational models, and, consequently, collective action strategies subordinated to authority. Along with other factors, this generates effects of social fragmentation and depoliticization. The conception of the neighborhood underlying social policies has fostered atomization, competition, clientelist capture, and the limitation of community development capacities, leaving communities at a disadvantage in influencing the processes that affect them (Gimeno 2017).

From an alternative perspective, community strengthening (Montero 2010) proposes linking collective action processes to enhance community capacities, gain greater control over living conditions, and develop a shared community development project. This process, in turn, contributes to strengthening civil society by positioning committed participation and awareness‐raising as fundamental conditions for communities to engage more symmetrically and effectively. This modifies the mechanisms of subjectivity production (Montero 2010), enabling community self‐management processes that prioritize collective elaboration by community members without ceding this capacity to external agents from government institutions (Lefebvre 2009).

This perspective is considered “alternative” insofar as it departs from mainstream community psychology approaches that conceptualize psychosocial phenomena as discrete variables and address participation primarily as individual behavioral change. Instead, it adopts a relational and situated epistemology, understanding community processes as co‐produced within structural, territorial and identity‐based conditions. In this sense, the analytical lens is explicitly intersectional, recognizing that vulnerability emerges from the interaction of class, gender, ethnicity, territorialization and institutional power, rather than from any single dimension in isolation.

Zambrano and Henríquez (2019) argue that addressing community strengthening processes requires attention to the psychosocial community dynamics that emerge within the social fabric, are constructed by actors in a given territory, and mediate change and community empowerment. Among the dimensions they suggest addressing are: sense of community (Ayala 2024), social capital (Aedo et al. 2020), empowerment at different levels (Zimmerman 2000), leadership (Andrade 2013), and participation (Wiesenfeld 2016). Social capital is understood as resources that circulate within social networks and can generate well‐being and opportunities for those who are part of them (Lin 2001), specifically, resources such as information, reciprocity obligations, and the social control effect derived from the functioning of social norms (Coleman 2001). These resources can operate at the level of an individual's egocentric network (individual social capital), or through a group which is formed from the egocentric networks of the individuals that make it up (group social capital), or a collective, which could be a territorial community such as a rural agricultural or Indigenous community or a stable neighborhood community (community social capital) (Durston 2003).

At the individual and group level, information facilitates access to opportunities, and reciprocity functions as a source of social support. At the community level, information enables the functioning of community social norms, which, in turn, make collective action and community members’ control over internal dynamics possible (Durston 2003). The positive effects of social capital depend on the characteristics of the social networks that enable its formation and circulation (Herreros and De Francisco 2001). To achieve this, the structure or form of the network must have sufficient connectivity—meaning that all or most of its members are directly or indirectly connected—so they can access the resources that circulate within it (González‐Bailón 2006).

On the other hand, the sense of community is a subjective experience of belonging to a larger collective, being part of a network of mutual support relationships in which one can rely (Stewart and Townley 2020). McMillan and Chavis (1986) consider four aspects in this construct: (a) membership or a sense of belonging to a larger collective; (b) influence that can be exerted within this collective; (c) integration and satisfaction of needs by belonging to the community, commitment, and shared emotional bonds; and (d) the belief that members have spent time together, have shared and continue to share a history, common places, and similar experiences.

These aspects are associated with community empowerment, which refers to the articulated capacity to advance a collective well‐being project, where people are cohesive, organizations are interconnected, and there are links with external organizations and institutions beyond the communities. All of this should be mediated by a growing politicization of the understanding of reality by the community members (Sánchez 2024).

In the field of community psychosocial intervention, however, evidence shows that these dimensions have been addressed in isolation (Zambrano Constanzo et al. 2021), emphasizing how they behave in relation to specific interest groups within communities. This approach, which assumes an alleged independence of these dimensions, results in a fragmented understanding and analysis of community psychosocial dynamics, simplifying social reality and establishing causal relationships for identified issues. This negatively impacts the search for comprehensive and lasting solutions, as it fails to consider neighborhoods as knowledge‐producing agents.

In response to this, we propose a complex and integrated approach to these dimensions, assuming that reality presents itself as an indivisible whole, based on a network of interconnections that must be examined multidimensionally (Naidoo et al. 2017).

## 2 Participatory Research

Participatory approaches in research are used in various fields such as education, health, human rights, and disadvantaged groups, among others (Sandoval and Martínez 2021). This study approach facilitates mutual benefit between the community and academic partners, allowing the intersection of knowledge produced by academic frameworks with the communities’ own knowledge (Moosa‐Mitha and Wallace 2020).

Participatory research is a methodological perspective that enables the construction of situated knowledge for social actors to use in taking action within their community, transforming their living conditions, and fostering a progressive level of control over their environment (Montero 2009). In this sense, it is a methodological option that influences the community members’ ability to participate, organize, and improve their effectiveness in addressing collective challenges associated with enhancing their quality of life. This perspective aligns with the logic of community‐strengthening processes (Montero 2009).

To this end, the use of varied and creative methodologies is proposed, aimed at activating participation and involvement. These methodologies should also contribute to individuals’ ability to interpret reality and explore ways to change it (Mesa and Restrepo 2020). Beyond producing information, it also seeks to foster a transversal educational process that contributes to changing representations, attitudes, and behaviors, adding complexity to participation based on its effects—in other words, ensuring that participation helps generate new opportunities for further participation (Zambrano and Berroeta 2012).

In this study, we specifically adhere to participatory evaluation (Soler et al. 2014), which contributes to generating an integrated model for the participatory evaluation of community psychosocial dynamics. Participatory evaluation is a process that integrates the production of mixed‐methods data while increasingly incorporating participatory processes. This allows for the establishment of a baseline for decision‐making and the initiation of coordination processes between community and institutional actors, with the aim of strengthening the dimensions that emerge as critical for development and well‐being (Zambrano Constanzo et al. 2021).

## 3 Research Problem and Gap in the Literature

Existing literature in community psychology has addressed various dimensions such as sense of community, social capital, and empowerment individually (Ayala 2024; Aedo et al. 2020; Zimmerman 2000). However, there is limited integration of these dimensions in the analysis of psychosocial dynamics in vulnerable urban neighborhoods. This theoretical and empirical gap hinders a comprehensive understanding of the factors that influence community cohesion and organization in contexts of structural inequality.

The issue addressed by this study lies in the need for an approach that considers these dimensions in an interconnected manner, allowing for a more complex reading of the psychosocial processes that shape community strengthening. Specifically, this study seeks to analyze how these dynamics are affected by sociohistorical factors, power relations, and processes of politicization within the studied neighborhoods.

This study contributes to filling this gap by providing an integrated analysis that articulates a sense of community, social capital, and empowerment within a participatory evaluation framework. By adopting this approach, we aim to generate applicable knowledge to design more effective and sustainable intervention strategies in vulnerable urban communities, thereby strengthening their capacity for agency and social transformation.

However, although previous studies have described participation or local community networks, most of them examine these dimensions separately rather than as a relational system. What remains insufficiently addressed is not whether communities possess resources or bonds, but *how* these capacities are activated, sustained or eroded in everyday life (Zambrano Constanzo et al. 2021). This study advances this gap by adopting an integrative approach that understands strengthening as a dynamic and situated process, rather than a static attribute of the territory, where practices, meanings, and relational conditions coproduce one another (Zambrano et al. 2024). Furthermore, the participatory methodological design does not only observe community dynamics but produces evidence that is returned to the community, enabling residents to identify leverage points for transforming their own organizational capacity. In doing so, the study contributes both conceptually and methodologically, by showing that strengthening emerges through interaction among multiple dimensions and not as an isolated construct.

Accordingly, this study is guided by the following central research question: How do sense of community, social capital, and empowerment interact in vulnerable urban neighborhoods, and to what extent do these psychosocial dynamics enable or constrain community strengthening within a participatory framework?

## 4 Context of the Study

The research took place in the urban belt of a medium‐sized city in southern Chile, marked by persistent socio‐spatial inequality, high rates of poverty and unemployment, and a sustained erosion of collective resources produced by territorial segregation and precarious forms of urban development. These Neighborhoods are historically located at the margins of investment in infrastructure, security, and public services, which intensifies residents’ exposure to environmental risk, institutional distance, and restricted opportunities for participation in decision‐making spaces.

These conditions have contributed to the reproduction of psychosocial vulnerabilities, affecting not only access to services but also trust in institutions, collective efficacy, and perceived agency. As documented in previous participatory diagnoses developed with local actors, the combination of structural precariousness and fragmentation in relational networks has weakened residents’ capacity for sustained community action, despite the presence of internal assets and historical forms of organization.

The three Neighborhoods included in this study are located within the urban‐poor area of the city and were prioritized due to their high levels of socioeconomic and housing vulnerability. B1 is situated in a riverside zone exposed to environmental risks associated with flooding and structural humidity. B3, although physically integrated into the urban layout, experiences marked socio‐spatial segregation due to its location adjacent to higher‐income residential sectors. B2 corresponds to an interstitial area on the margins of the consolidated city, with limited connectivity and restricted access to public services. The selection of these Neighborhoods followed the inclusion criteria defined in the ANID–FAPESP project, which considered: (a) location in territories institutionally recognized as highly vulnerable; (b) the existence of community actors willing to participate in a participatory evaluation process; and (c) previous collaboration links with university or territorial networks, which ensured feasibility and reciprocity in the production of situated knowledge.

## 5 Methods

This study adopted a multiple case design (De Salas et al. 2011) to comprehensively analyze community psychosocial dynamics in urban neighborhoods with high levels of vulnerability. This design allows for the understanding of community strengthening processes from a situated and territorially embedded perspective, in which each neighborhood is treated as a unique context shaped by its own history of organization, participation, and relational configurations. Consistent with the principles of community psychology, the study assumes that the community is not only a “field site” but an active agent in knowledge production (Montero 2009).

Since community strengthening involves the development of collective capacities for social transformation (Montero 2012), a mixed and participatory methodology was implemented to capture both the subjective dimensions of belonging and the structural configuration of the networks that sustain solidarity and cooperation. This methodological triangulation not only enriched the interpretation of community dynamics but also promoted co‐construction of knowledge with community actors themselves (Zambrano and Henríquez 2019).

In methodological terms, this overarching question is analytically developed through three subsidiary questions:

a. What contextual and organizational features account for differences across neighborhoods with distinct participatory trajectories?
b. How do quantitative findings (network configuration and sense of community levels) intersect with the meanings and practices emerging from participatory evaluation?
c. To what extent does the participatory process itself contribute to generating conditions for community strengthening, rather than merely documenting existing dynamics?

The selection of the three Neighborhoods responded to methodological inclusion criteria rather than merely contextual description. Vulnerability was defined according to institutional classifications provided by the Ministry of Social Development and Housing (MIDESO), which designates territories with high socio‐residential precariousness, environmental risk, and limited access to public services as priority areas for social intervention. In addition, the Neighborhoods were part of the ANID–FAPESP territorial map of “critical urban zones”, characterized by cumulative disadvantage and low institutional responsiveness. The study therefore included only territories that met all three conditions: (a) formal recognition as highly vulnerable areas by public policy instruments; (b) the presence of existing grassroots or community actors willing to engage in the participatory process; and (c) previous or ongoing collaboration networks ensuring feasibility and reciprocity in knowledge production.

The fieldwork began with preliminary contact aimed at clarifying the objectives, scope, and meaning of the project in each Neighborhood, initially through the Neighborhood Councils and subsequently extending to territorial leaders and community organizations. Once the working framework was agreed — including the commitment to progressively share results and to develop actions that would strengthen participation — the formal linkage phase was initiated. This stage coincided with the final period of COVID‐19 restrictions, which meant that the first meetings were carried out virtually and mediated by “bridge figures” already connected to the territory.

After sanitary restrictions were lifted, the process moved to in‐person work through key informant interviews, group interviews, and participatory workshops, together with a systematic presence in community activities. Subsequently, and in line with the triangulation strategy, both the Sense of Community scale and the name‐generator questionnaire were administered in parallel with the participatory qualitative phase. Preliminary analyses were shared with Neighborhood boards and community teams to validate the emerging interpretations and guide their collective meaning‐making.

Finally, several instances of feedback and collective reflection were organized — including open community meetings and extended sessions with public officials — which allowed participants to contrast experiences, identify shared challenges, and discuss conditions for collective action. The process concluded with inter‐Neighborhood encounters among community leaders, where learnings derived from the evaluation were exchanged and collaborative agendas for continuity were developed.

The methodological sequence was structured so that each phase of fieldwork was linked to specific instruments and analytical purposes. During the initial linkage phase, in‐depth interviews with key informants helped to identify community histories, organizational trajectories and entry points for trust‐building. In the second phase, group interviews and participatory workshops enabled the co‐identification of relevant community dimensions and tensions, shaping collective interpretations of local dynamics. In parallel, the Sense of Community scale captured subjective belonging and perceived cohesion, while the name‐generator questionnaire mapped the structural configuration of relational networks and the circulation of resources. Finally, the feedback and reflection phase functioned as a space of validation and methodological accountability, in which findings were returned to residents to refine meanings and derive implications for collective action.

### 5.1 Participants

The selected neighborhoods were characterized by high social vulnerability, socio‐territorial segregation, and prior trajectories of community organization defined by the ANID–FAPESP project. The inclusion of community organizations and key actors responded to the need to analyze how social capital is configured and mobilized in local relational systems (Aedo et al. 2020; Lin 2001), and how these processes interact with empowerment and community strengthening (Zimmerman 2000).

A total of 240 residents completed the initial questionnaire battery; after excluding cases with missing data on key variables, the final analytic sample included 226 participants, of whom 36% lived in Neighborhood 1 (B1), 37% in Neighborhood 2 (B2), and 27% in Neighborhood 3 (B3). Most participants were women (62% in B1, 59.8% in B2, and 80.7% in B3), homeowners, and long‐term residents. Although there were no age differences across neighborhoods, participants in B3 reported a significantly longer residence time (*F* = 10.7, *p* < 0.001), consistent with stronger historical rootedness.

Two partially overlapping groups took part depending on the instrument administered. Sense of Community was assessed among the 226 survey respondents, capturing the subjective experience of belonging and cohesion. In contrast, social capital was measured using a name generator questionnaire, applied to those most engaged in neighborhood life (egos). This subsample included 133 egos: 47 in B1, 45 in B2, and 41 in B3. Because network analysis incorporates both egos and alters, the reconstructed sociocentric networks included 166 nodes in B1, 156 in B2, and 158 in B3.

Recruitment followed a participatory strategy facilitated by community leaders, neighborhood associations, and local organizations, ensuring the inclusion of both formal and informal actors (leaders, connectors, caregivers, emergent organizers). This design made it possible to examine simultaneously the psychological experience of community and the relational infrastructure that sustains collective action—an uncommon combination in studies of urban vulnerability.

### 5.2 Design, Phases and Procedures

The study involved three interconnected phases. The first phase, exploratory in nature, consisted of preliminary meetings with neighborhood actors and group interviews aimed at reconstructing collective memory, mapping key relationships, and identifying ongoing community processes. Throughout this phase and transversally to the entire study, the research team also engaged in community life through participation in local events and the collaborative organization of informal spaces (e.g., garden restoration, murals, children's celebrations, cultural/recreational activities). This sustained presence strengthened trust and horizontal dialog, creating conditions for ethically grounded and contextually meaningful data production.

The second phase involved the diagnostic stage, comprising the application of quantitative instruments: the Sense of Community scale and the name generator questionnaire.

The third phase consisted of collective interpretation, where preliminary findings were returned to residents through facilitated workshops that enabled validation, refinement, and reinterpretation of the data from the perspective of local meanings and practices.

### 5.3 Techniques and Instruments

Data collection combined qualitative and quantitative techniques to develop a multidimensional view of psychosocial dynamics (Naidoo et al. 2017). Participant observation supported the recognition of organizational practices, reciprocity, and leadership emergence. Semi‐structured interviews with key informants deepened interpretations around participation, identity, and local governance of care. Group interviews and participatory techniques helped create dialogic spaces for critical reflection and collective problematization (Wiesenfeld 2016).

The Sense of Community Scale was originally developed by Sánchez‐Vidal (2001) to assess neighborhood perception and sense of community, drawing on Sarason's (1974) seminal conceptualization and subsequent contributions from other authors including Gil Lacruz et al. (1996) and Davidson and Cotter (1986). The original version consists of 18 items rated on a seven‐point Likert scale measuring the degree of agreement (1 = “strongly disagree” to 7 = “strongly agree”) with statements related to territorial identity (“I feel rooted in this place”), neighbor interaction (“I frequently interact with and know my neighbors”), and interdependence or mutuality (“I believe we all depend on one another”). For the present study, a previously adapted version validated for the Chilean context was used. A confirmatory factor analysis conducted with data collected from residents of neighborhoods in Temuco indicated that a two‐factor model provided a better fit than the three‐factor solutions tested in earlier versions (Guitart and Vidal 2012). These two factors correspond to interdependence with neighbors (Cronbach's alpha = 0.82) and neighborhood identity (Cronbach's alpha = 0.83) (Trujillo et al. unpublished manuscript). The name generator questionnaire allowed for the identification of supportive relational ties (Wasserman and Faust 2013). Although the data were collected egocentrically, the analysis was sociocentric, reconstructing complete neighborhood networks to examine capital distribution, centrality, bridging actors, and structural cohesion. All statistical analyses, including confirmatory factor analysis and descriptive comparisons, were performed using Stata 17 (StataCorp 2021), while network metrics and visualizations were computed with UCINET 6 (Everett and Borgatti 2012).

### 5.4 Triangulation and Data Integration

The analytical strategy relied on complementary triangulation. The Sense of Community scale captured subjective belonging, network analysis revealed the structural dimension of social capital, and qualitative material contextualized and interpreted both, linking metrics to lived experience. The integration occurred not only at the analysis stage but also through collective interpretation in workshops, ensuring ecological validity and alignment with situated knowledge.

Qualitative material (interviews, observation notes, and workshop dialogs) was examined through thematic analysis, identifying patterns that complemented and deepened the understanding of network configurations and their links to empowerment and community capacity.

Beyond technical triangulation, the integration of methods in this study follows an epistemic logic grounded in coproduction. Quantitative indicators (sense of community levels and network structure) provided a structural map of cohesion and relational density, while qualitative material illuminated the symbolic meanings, historical trajectories, and lived practices that sustain or erode these ties. This analytic complementarity made it possible to link network form with community meaning‐making, rather than treating them as separate dimensions.

Integration also occurred at the procedural level, through iterative and dialogical interpretation with residents during community workshops. This approach aligns with what Montero (2009) conceptualizes as “epistemic empowerment”: the capacity of participants not only to contribute information, but also to reinterpret it, dispute its implications, and situate it within their own forms of agency. In this sense, the mixed‐methods design is not ancillary to the participatory approach but constitutive of it, producing knowledge that is simultaneously descriptive, interpretive, and transformative.

### 5.5 Qualitative Analysis

The qualitative material was analysed through a thematic approach informed by participatory and situated criteria. An initial round of open coding was conducted to identify recurring meanings and tensions emerging from interviews, observation notes and workshop dialogs. These codes were subsequently refined through axial grouping to articulate broader interpretive categories linked to the dimensions under study (sense of community, social capital, empowerment and participation). Analytical rigor was ensured through two complementary procedures. First, theoretical saturation was applied as a criterion for closing the coding process, meaning that no new interpretive categories emerged in the final stages of analysis. Second, intercoder agreement was reached through collaborative review sessions among the research team, where coding decisions were compared and discrepancies resolved. Validation was further strengthened through community feedback assemblies, in which preliminary categories were collectively examined, reinterpreted and situated within local meaning frameworks. This iterative movement between analysis and community review ensured a reflexive and non‐extractive interpretive process grounded in participants’ lived perspectives.

### 5.6 Ethical Considerations

This study was approved by the Scientific Ethics Committee of Universidad de La Frontera (Folio No. 133/20). Informed consent was requested from participants for each technique used, ensuring the voluntary and confidential nature of their participation. Participants’ physical and mental well‐being was safeguarded, allowing them to refuse participation partially or entirely without repercussions. Additionally, confidentiality was protected through anonymization processes.

Following the principles of community psychology, the research findings were shared with the community and other relevant actors in agreement with participants, aiming to guide decision‐making and contribute to the collective well‐being of the studied neighborhoods.

It is important to highlight that this study's methodological design not only allows for the description of community psychosocial dynamics but also facilitates the generation of empowerment and organizational strengthening processes. The combination of mixed methods and participatory approaches addresses the challenges of community empowerment in vulnerable contexts by fostering a comprehensive and situated understanding of social transformation processes in these neighborhoods.

## 6 Results

The results are presented across three interrelated analytical dimensions of community strengthening: (a) sense of community, as the affective bonds, place‐based belonging, and territorial identity; (b) social capital, expressed in the structure and circulation of relational resources; and (c) empowerment, examined at individual, organizational, and community levels, including patterns of interaction with public policy. Finally, an integrated analysis is provided to illustrate how these dimensions operate simultaneously as both resources and constraints in the three neighborhoods studied.

In terms of socio‐environmental description, B1 is located near a river, B2 is situated in a hilly area, and B3 is adjacent to residential neighborhoods. In the latter, a perimeter fence prevents movement toward these areas and isolates it from the rest of the sector. All three neighborhoods are urban settlements that emerged between the 1980s and 1990s. B1 was formed through land occupations near the river by young families organized as a group of squatters. It consists of basic housing with progressively developed services. B2 is a subdivided area where residents purchased plots and gradually built their homes through self‐management. Some houses were acquired through state housing subsidies. Basic services were obtained through collective and sustained efforts toward urbanization. B3 was established through the relocation of informal settlements, with housing subsidies granted to the residents. The neighborhood was formed in stages, bringing together groups relocated from informal settlements in Temuco, some of which had prior community organization, while others came from outside the city. According to early residents, these latter families were perceived as more problematic. In all three neighborhoods, urbanization has been progressively developed, actively driven by community organization.

### 6.1 Sense of Community

In B1, there is a strong sense of community and a history of solidarity and mutual aid among neighbors, especially among older adults. However, more effort is needed to foster integration and promote stable activities that engage all residents inclusively. Despite this, community organizations such as the Neighborhood Council play a key role in promoting participation and social cohesion. The emotional bonds shared among residents, strengthened by time and mutual acquaintance, generate a sense of affection and belonging to the community. These elements are reflected in the following quote:

I think it's the fact that we have been living here for so many years. Let's put it this way: when we arrived, we were young, and now we're not so young anymore. So, we already know our neighbors (…) In the end, you become fond of the place where you live, and as the years go by, it gets even stronger—you get more sentimental.

(E‐C‐FIC3‐1)

In B2, the Neighborhood Council is led by its president, who is responsible for organizing activities and addressing community issues, generating satisfaction among nearby neighbors. However, many residents do not participate in decision‐making and are unaware of activities, which limits their influence in the community. Support networks are present during difficult times, but they are spontaneous rather than organized. The pandemic has led to changes in interactions, and some resistance has emerged due to distrust. Some neighbors display strong emotional ties, while others remain distant. In B2, a sense of community is observed among organized groups, demonstrated through solidarity and mutual aid actions. Table 1 and 2:

People are somewhat united, yes. e.g., when a person in need passes away, a small collection is organized: food or a bit of money.

(E‐C‐VIC2‐1)

**Table 1:** Phases, Instruments, and Analytical Purposes in the Fieldwork Process.

Phase	Instruments	Purpose
Linkage and trust‐building	Key informant interviews	Understand community history, relational anchors
Participatory diagnosis	Group interviews and workshops	Co‐construct meanings and identify tensions
Parallel triangulation	Sense of Community scale + name generator	Capture belonging and network structure
Collective reflection	Feedback assemblies	Validate findings and derive implications

**Table 2:** Sample composition by neighborhood and data collection method.

Neighborhood	SOC survey (*n*)	% Women (survey)	Name‐generator egos (*n*)	Sociocentric network nodes (*n*)
B1	81	62.0	47	166
B2	84	59.8	45	156
B3	61	80.7	41	158

*Note:* Survey percentage estimates derived from the final analytic sample of 226 participants.

In B3, the sense of community is characterized by a shared belonging to a larger collective and a network of mutual support relationships. This sense of community motivates certain groups of older adults to work for, with, and alongside the community. Activities such as neighborhood clean‐up days, aid campaigns, and children's workshops are organized, and traditions and celebrations of the neighborhood are revived. However, these interactions are concentrated within a small group and do not include young people, who primarily use public spaces such as the sports court and playground. There is concern about issues of violence and alcohol and drug consumption among young people, although it has been noted that public alcohol consumption is more associated with a specific group of adults. Neighbors highlight the community's solidarity and unity during times of hardship and the mutual support provided in difficult situations. In B3, a sense of community is evident, particularly among adults and older adults who have shared daily life in the neighborhood since its inception and continue to support one another.

It has always been a very united community. For example, when someone passed away, everyone would try to help the family. If something happens to someone, everyone comes together to help.

(E‐C‐EIC1‐1)

The results of the Sense of Community Scale indicate that all three neighborhoods exhibit generally high levels of neighborhood identity and interdependence with neighbors. Nonetheless, statistically significant differences were found in identity levels across the territories (*F* = 6.04, *p* = 0.003), with B3 showing higher territorial rootedness and identification with the neighborhood (*M* = 5.8, SD = 1.4) compared to B1 (*M* = 5.4, SD = 1.5) and B2 (*M* = 4.95, SD = 1.7). In contrast, interdependence levels were similar among the three territories, with slightly higher averages in B3 (*M* = 6.7, SD = 0.55) than in B1 (*M* = 6.57, SD = 0.72) and B2 (*M* = 6.56, SD = 0.9).

As shown in Table 3, the distribution of scores is not uniform across the three Neighborhoods, but reflects their distinct organizational histories and levels of collective cohesion, which is consistent with the qualitative material presented in the following section.

**Table 3:** Descriptive statistics of Sense of Community (Sánchez‐Vidal 2001) by neighborhood.

	B1	B2	B3	
	*N* = 81	*N* = 83	*N* = 62	
Length in the neighborhood (mean, SD)	19.9 (10.8)	19.8 (12.3)	28.3 (14.5)	*F* = 10.7, *p* < 0.001
Neighborhood identity (mean, SD)	5.5 (1.33)	5.32 (1.4)	6.1 (1.15)	*F* = 6.04, *p* = 0.003
Interdependence with neighbors (mean, SD)	5.6 (1.21)	5.45 (1.29)	5.71 (1.06)	*F* = 0.88, *p* = 0.4175

*Note:* Higher scores indicate stronger neighborhood identity and interdependence.

*Interpretive note*. B3 presents the highest levels of both identity and interdependence, consistent with its longer history of stable collective organization. B2 displays intermediate scores aligned with intermittent participation, while B1 shows comparatively lower averages, reflecting recent organizational fragmentation and weaker relational cohesion.

### 6.2 Social Capital

Regarding individual social capital in B1, based on the social network analysis carried, we note that only a small group of people has contact with others, limiting their access to resources such as information and reciprocal relationships. To analyze how connected a person is in the network, we include the centrality measure, which indicates the number of direct ties a node has with others. The average degree centrality is low, reaching 1.32. However, links are formed between people with higher centrality and leadership within the neighborhood. These social capital resources mainly circulate within neighborhood support networks, providing information about procedures and access to government resources.

In terms of group social capital, resources circulate primarily within cohesive groups of connections. To identify cohesive groups, cliques were used as a measure of social network analysis defined as a set of at least three nodes directly connected to each other. In addition, N‐cliques are identified, which are subgraphs in which the largest geodesic distance between any pair of nodes is not greater than 2. In the analyzed network there are 3 cliques and 38 N‐cliques. These groups are formed around spaces managed by community leaders, with whom people have closer ties and share common interests. The Neighborhood Council is one of the main groups concentrating community activities, yet a lack of active participation is observed due to limited awareness of its actions and challenges such as geographical distance or lack of access to communication technologies.

Other spaces for participation in the neighborhood include senior groups, churches, and cultural and environmental organizations. However, there is a notable lack of communication and connection among these groups, possibly due to differing objectives and values.

**Figure 1:**
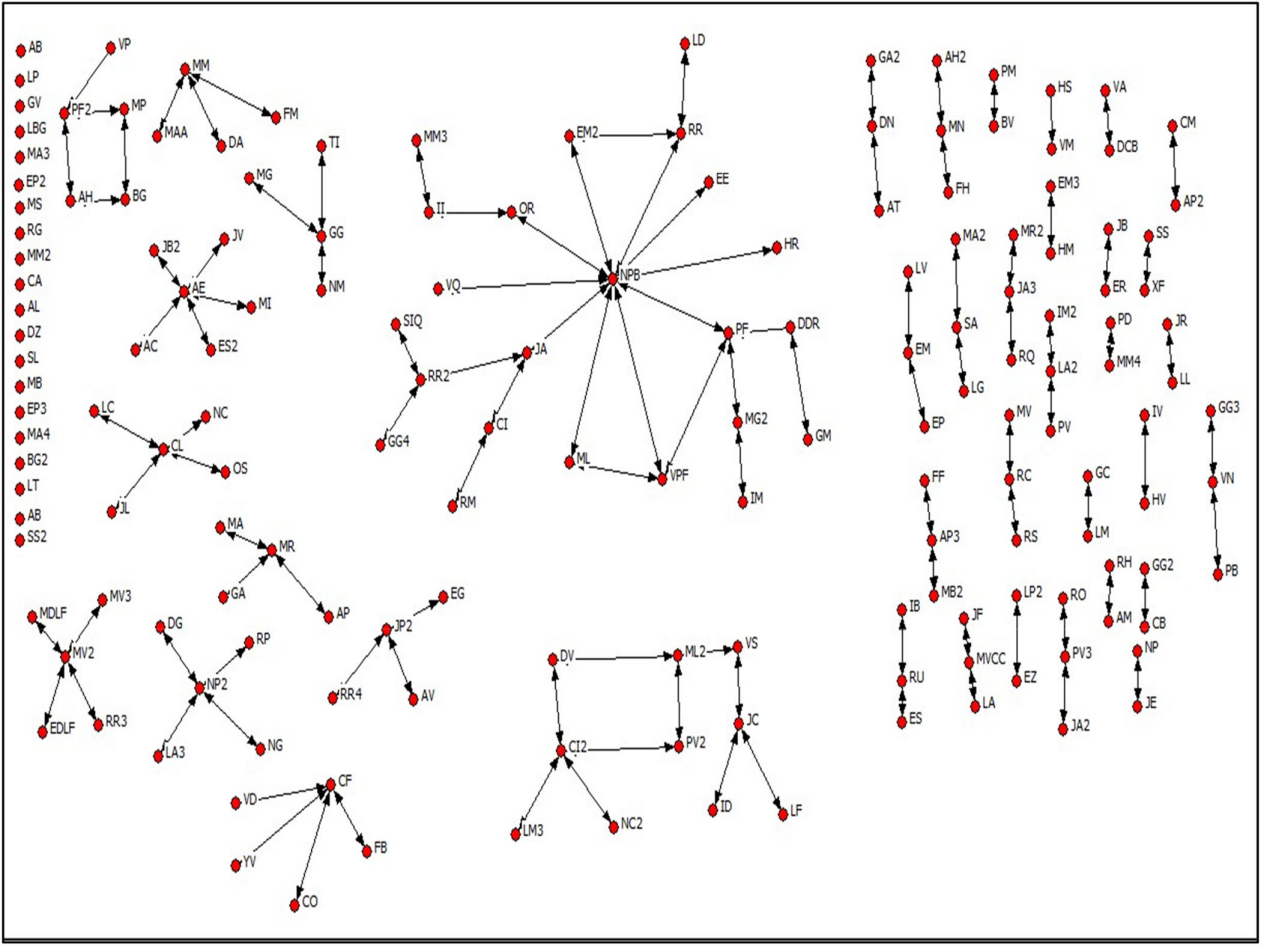
Social network of B1.

At the community level, social capital resources such as reciprocity and the generation of public goods through social norms exist. However, for social capital to be a true asset for the community, high connectivity between individuals is required. To analyze connectivity, we consider two network measures: density and components. Density is the ratio of existing to possible links in the network, based on the number of nodes within it. Density values range from 0 (there are no direct links between any network nodes) to 1 (all network nodes are directly connected to each other). Components are subsets of the network, each node of which is directly or indirectly connected. In relation to B1, the social network it is presented in the Figure 1.

Connectivity in social network of B1 is very low, with a network density of 0.008. The largest component of the network consists of 23 people, representing 13.9% of the total network (166 people). This indicates that most people are not directly linked to one another. Nonetheless, information can circulate among the neighborhood's female leaders, who hold more influential positions and could act as distributors of information throughout the area.

**Figure 2:**
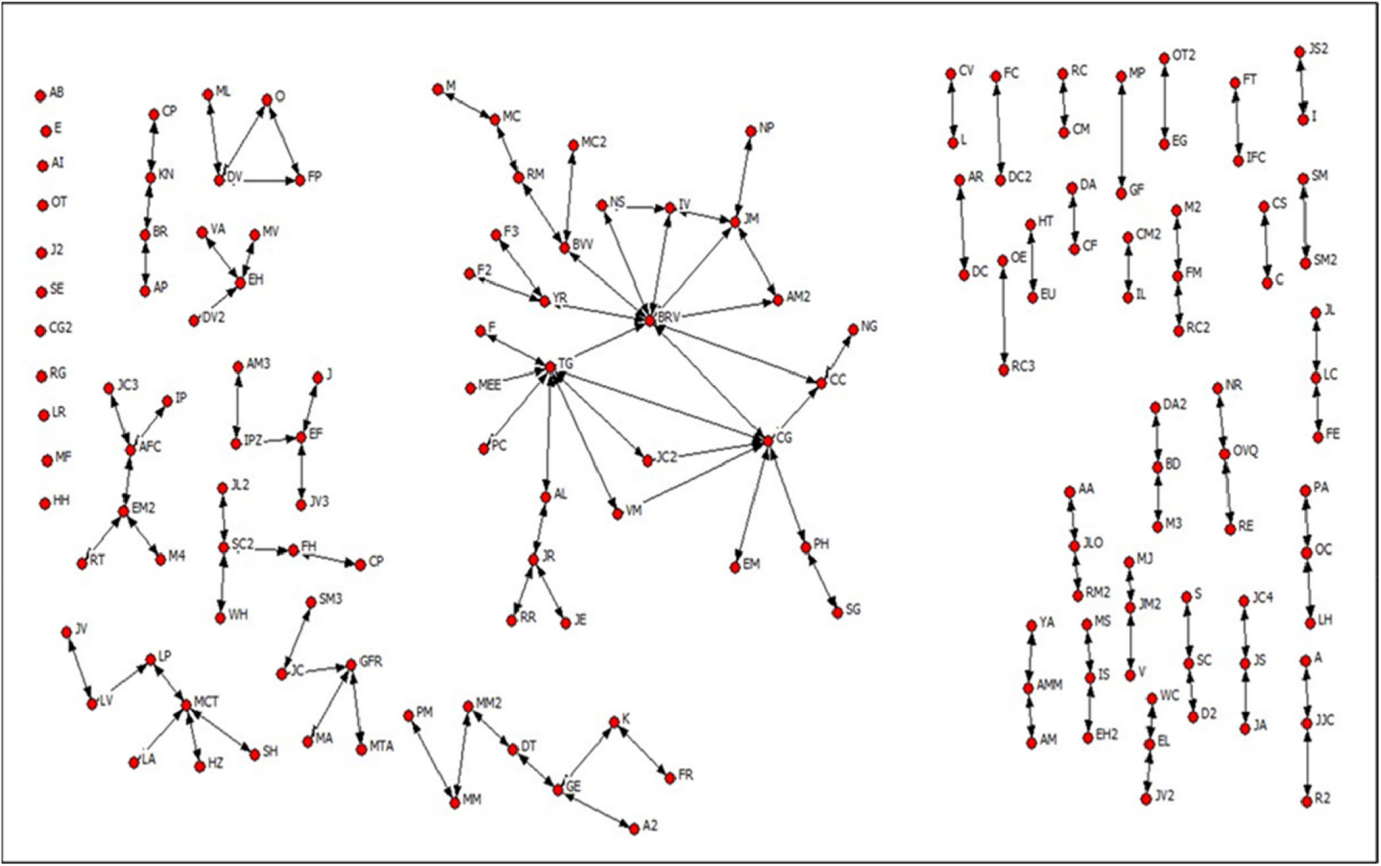
Social network of B2.

The social network of B2 it is presented in the Figure 2.

In B2, only a small group of people maintains contact with others, limiting access to resources such as information and reciprocal relationships. The average degree centrality is 1.48. Regarding group social capital, 8 cliques and 45 N‐cliques have been identified. This means that resources such as information and mutual aid circulate among a few people who form more cohesive relationships with one another than with the rest of the network.

Additionally, several organizations exist within the neighborhood, such as the Neighborhood Council, a senior citizens’ group, and a women entrepreneurs’ group, where social capital resources are exchanged. However, a lack of communication and cooperation between these groups is observed, possibly due to differing goals and values. Participation is primarily seen among adult women and elderly women in these organizations.

At the community level, social capital resources such as reciprocity and the creation of public goods based on social norms may exist. However, most individuals are not directly connected. The network density is 0.010. The largest network component consists of 30 people, accounting for 19.2% of the total network (156 people). This hinders the flow of information regarding compliance with social norms and the formation of expectations about adherence. In contrast, information can circulate among individuals in central community positions who act as distributors of information.

**Figure 3:**
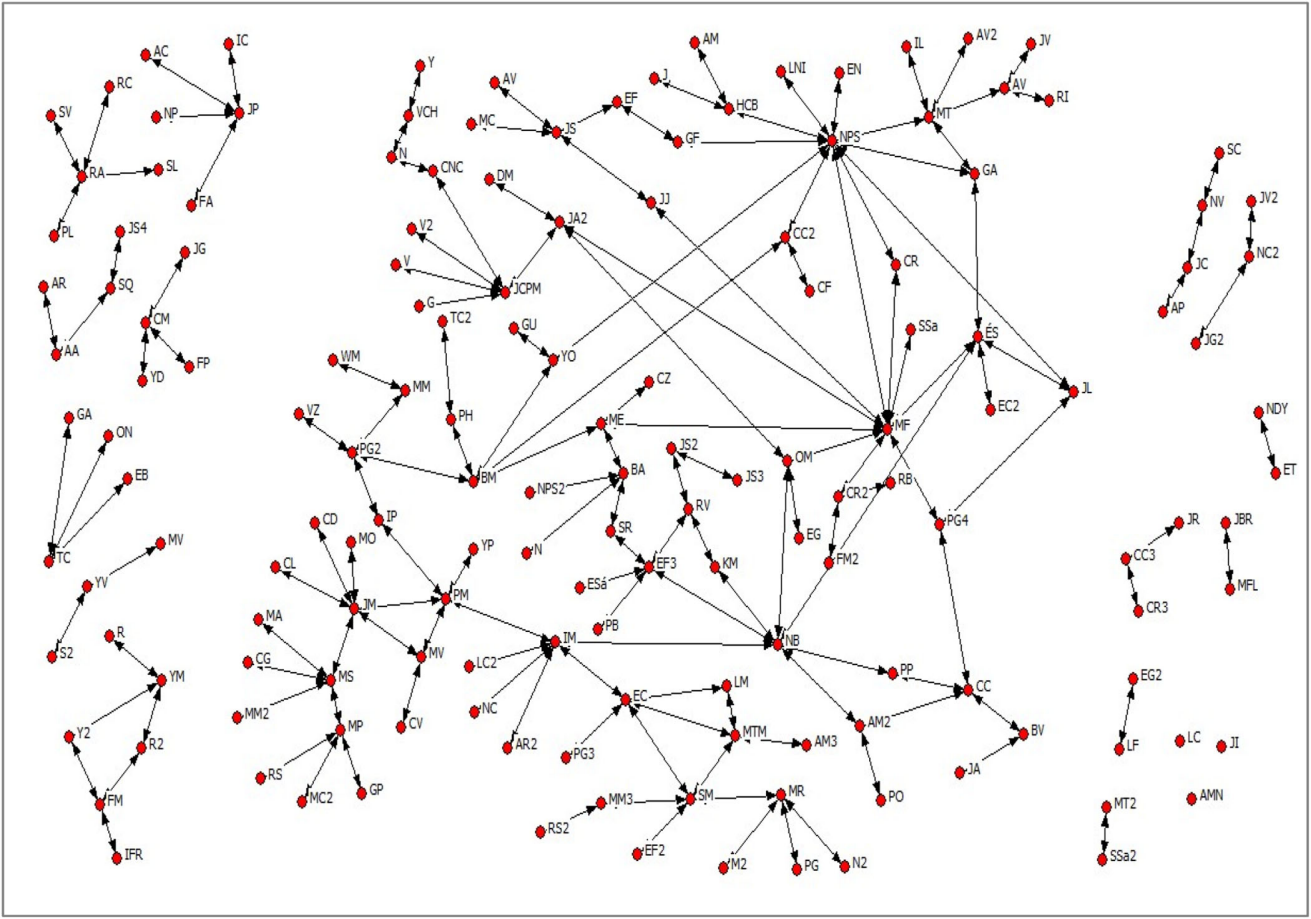
Social network of B3.

The social network of B3 it is presented in the Figure 3.

In B3, there is a higher level of connection among people in the neighborhood compared to B1 and B2. As a result, B3 residents have more contact with others and greater access to resources such as information and mutual support. The average degree centrality is 2.03. Individuals with the most central positions in the network, due to their numerous direct links with others, act as distribution channels for social capital resources.

Additionally, cohesive groups are present in the neighborhood, characterized by strong bonds among their members. Specifically, there are 6 cliques and 72 N‐cliques. In addition to social groups, community organizations such as the Neighborhood Council and sports clubs also generate group social capital. These organizations strengthen interpersonal relationships and contribute to the community's overall functioning.

Yes, a lady brought us together. The one who acted as president gathered us, summoned us to the community center, and that's how we met (…) From different streets, yes (…) she called us to the community center, and that's how we met. Regarding her participation in a(E‐C‐EIC3‐1). church, she states: We already knew each other before, yes. The church was established after we arrived here.

(E‐C‐EIC3‐1).

At the community level, social capital resources can be found. The network density is 0.013. This is a very low density value, similar to that of B1 and B2, considering, as previously noted, that a density value of 1 means that all nodes are connected, and 0 means that there is no connection between any nodes. In B1, B2, and B3, there is an absence of ties between most nodes, which hinders the formation of community social capital. However, despite the low density of B3's social network, it has some structural characteristics that favor greater connectivity compared to B1 and B2. One relates to the number of cliques in B3. Another relates to the configuration of its components. The largest network component consists of 106 people, representing 67% of the total network (158 people). However, despite the social network's characteristics, some factors hinder the flow of information and the formation of expectations necessary for enforcing social norms. Although shared social norms exist, there is no effective collective capacity to ensure their enforcement in all cases, leading to occasional issues within the community.

### 6.3 Empowerment

At the individual level, empowerment is primarily concentrated in a small group of community leaders, most of whom are adult or older women with long‐standing trajectories of neighborhood involvement. These leaders have developed management skills, know how to navigate bureaucratic procedures, and mediate relations with institutions. However, this empowerment is not widely distributed among other residents, which limits the emergence of new leadership and hinders community‐level agency.

At the organizational level, the three neighborhoods count on active community organizations (mainly the Neighborhood Council, senior citizen groups, and women's associations). Nevertheless, their internal effectiveness varies. In B1, leadership has a more reivindicative and rights‐oriented profile, oriented toward demanding structural changes and addressing historical grievances. Participation, however, does not extend widely across the neighborhood, making mobilization dependent on a small set of committed actors. In B2, organizational empowerment operates through a more pragmatic and clientelistic pattern of engagement, centered on negotiating access to concrete resources rather than on political or structural transformation. In B3, organizational structures are more stable and capable of managing projects, but still show limited participation beyond adult and elderly women.

At the community level, empowerment remains constrained. Although there are forms of collaboration and localized solidarity, there is not yet a collectively sustained capacity to influence decision‐making, construct shared community agendas, or articulate neighborhood‐based advocacy beyond organizational intermediaries. This translates into what may be described as an empowerment structure that is functional at the organizational level but not yet structural at the community level. This finding will be further developed in the Discussion section, where the implications for strengthening community agency are examined in depth.

### 6.4 Relationship with Public Policy

Patterns of interaction with public institutions also differ across the three neighborhoods and are closely tied to the type of empowerment that has been consolidated. In B1, leaders adopt a reivindicative, rights‐based stance toward the State, rooted in a history of distrust and perceived exclusion from decision‐making. Engagement is oriented toward structural change rather than transactional gain, although it remains concentrated in a small group of leaders. As one participant noted: *“Without a doubt, it's political clientelism. Otherwise, projects don't come through. If you don't align with the politician, you don't get the projects… But because we don't align politically, we've been left out of many projects.”* (E‐C‐FIC2‐1). This critical positioning shows elements of political empowerment at the leadership level, but limited diffusion to the broader community base.

In B2, the relationship with public institutions follows a more functional and instrumental pattern. Support is negotiated pragmatically with whichever authority responds to local needs, privileging access to resources over collective deliberation or institutional accountability. As a resident explained: *“It's not about a party, but rather about a council member or someone in politics who genuinely supports us—not just says they will, but actually helps with tangible things.”* (E‐C‐VIC3‐1). This enables resource circulation but reinforces dependency and does not foster a shared political project at the community scale.

In B3, institutional presence is more visible, particularly through social programs and municipal interventions. However, participation remains consultative rather than co‐governed, and organizations tend to operate as recipients of services rather than co‐producers of territorial decision‐making. Weak institutional coordination further reduces opportunities for structural community influence.

Across all three cases, community–state relations rely heavily on a small group of mediating leaders—mostly women—who act as brokers between residents and public institutions. While this leadership brokerage enables short‐term management and access to benefits, it also concentrates political competencies in a few individuals and limits the emergence of collective political agency throughout the neighborhood.

### 6.5 Integrated Analysis: Psychosocial Dynamics as Both Resource and Limitation

The integrated analysis of the three dimensions shows that psychosocial dynamics simultaneously operate as sources of strength and as constraints for community development.

As a resource, the neighborhoods display strong affective attachment (sense of community), local solidarity practices, and leadership‐based organizational capacity. Mutual aid is activated especially in times of crisis, and there is accumulated experience in managing relationships with institutions. In B3, in particular, denser relational structures (largest connected component) provide a stronger foundation for collaborative action.

As a limitation, these same dynamics remain concentrated rather than widely distributed. Participation revolves around a small circle of women leaders, which restricts intergenerational renewal and reduces inclusion of youth and men. The fragmentation of relational structures in B1 and B2, and the limited integration of organizations in all three neighborhoods, hinder the transition from organizational empowerment to broader community empowerment. Furthermore, interactions with public policy remain mediated and asymmetrical, reinforcing dependence on leadership rather than enabling community‐level Overall, the findings suggest that while there are important assets for community strengthening—particularly in affective cohesion and leadership‐based relational capital—these remain insufficient to generate structural empowerment at the community level. In the following discussion, we examine how the interplay between sense of community, social capital, and empowerment can help explain these patterns, and what conditions are necessary to shift from organizationally concentrated leadership to wider, community‐level agency capable of sustaining long‐term transformation. co‐governance.

## 7 Discussion and Conclusions

The findings provide an integrated understanding of the psychosocial dynamics observed in the three neighborhoods, showing that sense of community, social capital, and empowerment do not operate as isolated dimensions but as interdependent processes shaped by each territory's organizational and relational trajectories. Consistent with previous literature (McMillan and Chavis 1986; Stewart and Townley 2020; Lin 2001), affective belonging and solidarity‐based networks constitute a relevant foundation for collective action. However, as shown in these cases, their transformative potential is constrained by fragile organizational structures and low levels of politicization.

Regarding empowerment, it is largely concentrated in individual leaders who hold significant management skills and established ties with external actors. While this constitutes an important community resource, it also becomes a limitation to the extent that it does not translate into collective empowerment or into the structural strengthening of local organizations (Zimmerman 2000; Montero 2012). This tension—between functional empowerment centered on leadership and broader community‐based empowerment—reproduces asymmetrical forms of participation and keeps agency encapsulated within a small group of interlocutors (Zambrano Constanzo et al. 2021).

The analysis of social capital shows that it is mainly mobilized through bonding ties, reinforcing internal reciprocity and mutual aid, yet with limited development of bridging capital, which could connect the neighborhoods with a broader institutional ecology through logics of co‐responsibility rather than dependence. When relations with public policy are structured through instrumental or clientelist dynamics—as more clearly observed in B2 and partially in B3—the capacity to question or reshape the way the State intervenes in the territory becomes restricted (Berroeta et al. 2019). In B1, by contrast, a rights‐claiming and reivindicative orientation is present, although it has not yet expanded to the broader community base due to weak generalized participation.

A distinctive contribution of this study lies in that the methodology did not merely document community dynamics but also activated empowerment conditions through processes of coproduction of knowledge, positioning the community as an epistemic subject rather than as a passive object of study. The participatory design—based on observation, collective reflection, and continuous feedback—functioned simultaneously as a lens and as a strengthening device, enabling mutual recognition, internal reorganization, and expanded agency. This aligns with perspectives that understand participatory inquiry as a political, rather than solely technical, process (Montero 2009; Zambrano and Berroeta 2012).

The results suggest that the sustainability of community strengthening requires moving beyond leader‐centered organizational models toward forms of empowerment distributed across the community base (Perkins and Zimmerman 1995), fostering bridging social capital, diversifying participation spaces, and enabling everyday mechanisms of politicization that allow communities to contest State intervention and coproduce territorial decisions. In parallel, rotational and intergenerational leadership transitions are needed to broaden agency and prevent the concentration of power in a small number of mediating actors.

In terms of projection, the experience analyzed offers transferability not as a universal model but as a methodological horizon, demonstrating that situated knowledge production can also produce agency and institutional reconfiguration. This opens a path for public policies that recognize communities not merely as recipients of intervention but as co‐producers of the public, expanding their political subjectivity and territorial sovereignty.

Finally, rather than a closure, these results point toward a strategic field of action: the need to move from communities managed by “intermediary leaders” toward communities capable of collectively disputing, co‐producing, and shaping the conditions of life in their territories. The sustainability of community strengthening depends not only on internal cohesion but also on democratizing power, expanding the political subject, and transitioning from assistance‐based logics to co‐responsibility as a transformative horizon.

## Ethics Statement

This study was reviewed and approved by the Scientific Ethics Committee of Universidad de La Frontera (Approval No. 133/20). All procedures were conducted in accordance with the ethical standards of the institutional and national research committee and with the 1964 Helsinki declaration and its later amendments.

## Consent

Informed consent was obtained from all individual participants included in the study. Participants were informed about the research objectives, the voluntary nature of their participation, and the confidentiality of their data. They had the right to withdraw at any time without consequences.

## Conflicts of Interest

The authors declare no conflicts of interest.

## Permission to Reproduce Material

This manuscript does not include copyrighted material from other sources requiring permission for reproduction. If any material had required permission, appropriate licenses would have been secured in advance.

## Data Availability

The data supporting the findings of this study are available from the corresponding author upon reasonable request. Due to the participatory and community‐based nature of the research, data are not publicly available to protect participant confidentiality and community agreements.
